# Sleep Health and Obstructive Sleep Apnea Risk in Portuguese Firefighters: Preliminary Baseline Findings From a Prospective Cohort

**DOI:** 10.7759/cureus.103828

**Published:** 2026-02-18

**Authors:** Carolina Da Silva Alves, Luís Caldeira, Patrícia Ribeiro, António Osório, Paulo Longo, Patrícia Dâmaso, Ana Macedo, Luísa Lopes, Cecília Longo

**Affiliations:** 1 Pulmonology Department, Hospital Professor Doutor Fernando Fonseca, Lisbon, PRT; 2 Research, Chama Saude Health Association, Lisbon, PRT; 3 Research, Linde Saude, Lisbon, PRT; 4 Clinical Sales Specialist, Linde Saude, Leiria, PRT

**Keywords:** fire fighters, obstructive sleep apnea (osa), occupational health, polysomnography, sleep disorders

## Abstract

Background and objective

Firefighters face irregular schedules, night operations, and fragmented sleep, placing them at risk of sleep disorders and operational fatigue. Despite growing international evidence, sleep health data on Portuguese firefighters are scarce. In this study, we aimed to report baseline findings from the Associação Chama Saúde Initiative, a nationwide prospective cohort designed to characterize sleep health, obstructive sleep apnea (OSA) risk, and diagnostic yield within a structured screening-to-diagnosis framework, as well as the operational sleep context among Portuguese firefighters.

Methods

Active-duty firefighters completed a structured baseline online questionnaire (n=1815), including demographic data, medical history, lifestyle factors, the Epworth Sleepiness Scale (ESS), the STOP-Bang questionnaire, and operational sleep variables. Participants screening with high risk of OSA proceeded to home sleep testing (HST) and Pulmonology referral. Outcomes included descriptive screening metrics, the feasibility of the diagnostic cascade, and early treatment uptake.

Results

The mean age distribution was skewed toward 30-49 years (63.4%), and 75% were male. BMI categorization indicated that 69% of participants were overweight or obese. Cardiovascular comorbidities were reported in 19%, and previous mental health diagnoses in 24%. Excessive daytime sleepiness (ESS >10) was present in 589 participants (32%). STOP-Bang identified a high OSA risk in 377 participants (21%). Operational sleep restriction was pronounced, with 59% reporting ≤4 hours of sleep during service, compared with 92% reporting ≥5 hours off duty. A structured diagnostic cascade was feasible at the national scale: 1815 participants were screened, 603 screened positive, 370 underwent HST, 228 were diagnosed with OSA, and 77 initiated continuous positive airway pressure (CPAP)/auto-adjusting positive airway pressure (APAP) treatment.

Conclusions

Preliminary baseline data indicate a substantial burden of sleep-related symptoms and elevated OSA risk among Portuguese firefighters, compounded by operational sleep restriction. Nationwide diagnostic implementation proved feasible, with early treatment uptake observed. Longitudinal follow-up will assess clinical outcomes and treatment adherence.

## Introduction

Firefighting demands sustained vigilance, rapid decision-making, and physical resilience under unpredictable and often hazardous conditions. Irregular schedules, emergency call-outs, and extended night operations compromise sleep opportunity and continuity, contributing to circadian disruption, fatigue, and increased vulnerability to sleep disorders [1-5]. Systematic reviews report a high prevalence of poor sleep quality, excessive daytime sleepiness, and sleep-disordered breathing among firefighters, with evidence linking sleep restriction to impaired reaction time, vigilance deficits, occupational accidents, and adverse cardiometabolic outcomes [2,6].

Obstructive sleep apnea (OSA) is of particular relevance to this occupational group. Studies indicate that 25 to 30% of firefighters may screen positive for OSA risk, with underdiagnosis exceeding 80% [3-4]. OSA and short sleep duration have also been associated with hypertension, obesity, metabolic dysfunction, and cardiovascular events, which are leading causes of duty-related morbidity and mortality among firefighters [7,8]. Despite expanding international data, similar evidence in Portuguese firefighters remains limited. Prior national studies have been small in scale, cross-sectional, or narrowly focused [9-11]. To address this gap, the ENASB study (Estudo Nacional da Apneia do Sono em Bombeiros) was developed as a nationwide prospective cohort integrating screening, diagnostic testing, and clinical referral.

This preliminary report aims to (1) describe the baseline characteristics and sleep screening findings of the cohort, and (2) report feasibility and early outcomes of the screening-to-diagnosis cascade for sleep-disordered breathing. Longitudinal outcomes and treatment compliance will be reported in future analyses.

## Materials and methods

Study design and population

This initiative involves a nationwide prospective cohort of active-duty Portuguese firefighters aged ≥18 years. Data collection began in June 2022 and remains ongoing, with planned completion in 2028. The present analysis includes baseline data collected between June 2022 and December 2025 (n=1815). Recruitment was conducted through national dissemination coordinated by the Portuguese Firefighters League and participating fire departments. Invitations were distributed electronically via institutional mailing lists and internal communication channels. Participation was voluntary and based on self-enrolment through a secure online platform. The total number of firefighters who received the invitation could not be precisely quantified due to multi-channel dissemination. This project is carried out through a collaboration between Chama Saúde (a medical non-profit organization serving firefighters) and Linde Healthcare, with the institutional support of the Portuguese Firefighters League. Ethical approval was granted by the Chama Saúde Ethics Committee in May 2022.

Screening procedures

Participants completed baseline sleep screening with the Portuguese-validated versions of the STOP-Bang questionnaire [12] and the Epworth Sleepiness Scale (ESS) [13]. The STOP-Bang is a validated eight-item screening tool for OSA, incorporating Snoring, Tiredness, Observed apneas, high blood Pressure, BMI, Age, Neck circumference, and Gender. In this study, high OSA risk was defined as a STOP-Bang score ≥4. The ESS is an eight-item validated questionnaire assessing subjective daytime sleepiness (score range: 0-24), with ESS ≥10 considered indicative of excessive daytime sleepiness.

Eligibility for sleep test and diagnostic workflow

Eligibility for home respiratory polygraphy (type III home sleep testing, HST) was operationalized according to predefined criteria. Participants qualified for HST if at least one of the following conditions was met:

(1) Both STOP-Bang (≥4) and ESS (≥10) were positive; (2) one positive screening test (STOP-Bang or ESS) in the presence of medical risk factors and/or sleep-related symptoms or drowsy driving; (3) sleep-related symptoms combined with cardiometabolic risk factors, regardless of screening results; and (4) prior OSA diagnosis with persistent symptoms and no current positive airway pressure (PAP) therapy.

Medical risk factors included cardiovascular, cerebrovascular, and metabolic comorbidities. Sleep-related symptoms included witnessed apneas, gasping, and habitual snoring. The sole exclusion criterion was current PAP therapy. OSA diagnosis following HST was based on the respiratory event index (REI), with REI ≥5 events/hour considered diagnostic and severity classified according to standard thresholds. Participants with REI <5 events/hour but persistent clinical suspicion were referred for specialist sleep consultation for further evaluation, including consideration of in-laboratory polysomnography (Type I or Type II) when clinically indicated.

Outcomes

The primary outcomes were the baseline sleep screening metrics, including STOP-Bang risk distribution and ESS scores. Secondary outcomes included operational sleep restriction during and outside duty periods, as well as the diagnostic cascade from screening to HST, OSA confirmation, and treatment initiation.

Statistical analysis

Data were analyzed using SPSS Statistics version 30.0 (IBM Corp., Armonk, NY). Descriptive statistics were used to summarize baseline characteristics and screening metrics. Categorical variables were expressed as counts and percentages. Continuous variables were summarized as means and standard deviations (SD). As this preliminary report was descriptive in nature, no inferential analyses were performed.

## Results

Baseline characteristics

A total of 1815 active-duty firefighters completed the baseline questionnaire. Demographic and anthropometric characteristics of the cohort are summarized in Table 1. Demographic data were available for 1747 participants (96.2%), of whom 1311/1747 (75.0%) were male. Most participants were aged 30-49 years (1107/1747; 63.4%). Overweight (724/1747; 41.4%) and obesity (505/1747; 28.9%) were common. Together, overweight and obesity affected 1229 of 1747 participants (70.4%).

**Table 1:** Demographics and anthropometric characteristics Baseline demographic and anthropometric characteristics of active-duty Portuguese firefighters participating in the Chama Saúde initiative. Missing data were not excluded BMI: body mass index

Variable	n/N	%
Total screened	1815	100
Demographic data available	1747/1815	96.2
Male	1311/1747	75.0
Female	436/1747	25.0
Age 30–49 years	1107/1747	63.4
Normal BMI	500/1747	28.6
Overweight	724/1747	41.4
Obesity (I–III)	505/1747	28.9

Health, lifestyle, and mental health

Self-reported comorbidities, lifestyle factors, and mental health conditions in the cohort are shown in Table 2. Cardiovascular comorbidities were reported in 340/1815 (18.7%) and mental health conditions (anxiety/depression/post-traumatic stress disorder (PTSD)) in 430/1815 (23.7%). Smoking (current or former) was reported in 972/1815 (53.5%). Prior OSA diagnosis was reported in 98/1815 (5.4%).

**Table 2:** Health, lifestyle, and mental health characteristics Self-reported comorbidities, lifestyle factors, and mental health conditions at baseline. Mental health conditions included anxiety, depression, and post-traumatic stress disorder OSA: obstructive sleep apnea

Variable	n/N	%
Cardiovascular comorbidity	340/1815	18.7
Mental health condition	430/1815	23.7
Insomnia diagnosis	226/1815	12.4
Current smoker	629/1815	34.7
Ex-smoker	343/1815	18.9
Prior OSA diagnosis	98/1815	5.4

Sleep screening and operational sleep

Sleep screening and operational sleep metrics are presented in Table 3. Excessive daytime sleepiness (ESS >10) was observed in 589/1815 (32.5%), and STOP-Bang identified high OSA risk in 377/1815 (20.8%). Sleep restriction during service was pronounced: 930/1815 (51.2%) reported ≤4 hours of sleep during service, compared with 1667/1815 (91.9%) reporting ≥5 hours off-duty. Shared sleeping quarters during service were reported by 1431/1815 (78.9%). These patterns are consistent with operationally driven sleep restriction rather than generalized insomnia, although causal inference cannot be established in this preliminary analysis.

**Table 3:** Sleep screening and operational sleep metrics Sleep screening instruments (ESS and STOP-Bang) and operational sleep characteristics during and outside of duty. Operational data were self-reported ESS: Epworth Sleepiness Scale

Variable	n/N	%
ESS >10	589/1815	32.5
STOP-Bang high risk	377/1815	20.8
≤4h sleep during service	930/1815	51.2
≥5h sleep off-duty	1667/1815	91.9
Shared dormitory	1431/1815	78.9
Sleep latency >30 min	564/1815	31.1

Screening-to-diagnosis cascade

**Figure 1:**
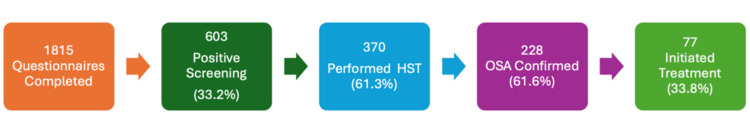
Screening-to-diagnosis cascade Flow of participants through the screening-to-diagnosis cascade from baseline questionnaire to treatment initiation within the Chama Saúde initiative. Values expressed as absolute numbers and percentages (%) relative to the previous step HST: home sleep testing; OSA: obstructive sleep apnea

A structured screening-to-diagnosis cascade was feasible at the national scale (Figure 1). Of the 1815 screened participants, 603/1815 (33.2%) screened positive, 370/603 (61.3%) completed HST, 228/370 (61.6%) were diagnosed with OSA, and 77/228 (33.8%) initiated CPAP/APAP treatment. Before screening, only 98/1815 (5.4%) reported an OSA diagnosis. Early treatment uptake was observed in 77/228 participants, with longitudinal compliance to be evaluated in follow-up analyses.

## Discussion

This preliminary analysis indicates substantial symptom burden and elevated screening-identified OSA risk in Portuguese firefighters, with a distinctive operational sleep profile marked by service-related sleep restriction. The proportion reporting excessive daytime sleepiness (32%) and screening-identified high OSA risk (21%) aligns with international findings [1,3], reinforcing sleep health as a key occupational determinant.

Underdiagnosis was pronounced: only 5% reported prior OSA diagnoses despite elevated screening positivity and diagnostic yield in the present cohort. Similar patterns of underdiagnosis have been documented in US cohorts [3,4], where structured screening programs identified a substantial proportion of previously unrecognized individuals at high risk for OSA or with subsequently confirmed disease. Early identification may mitigate downstream cardiometabolic risk and fatigue-related performance decrements, both relevant in firefighters, in whom cardiovascular events remain a major source of duty-related mortality [8].

Operational sleep metrics highlight an environment conducive to cumulative sleep debt. Reduced sleep during service, shared sleeping quarters, and prolonged sleep latency may contribute to circadian dysregulation, daytime sleepiness, and operational fatigue. These findings align with experimental work showing degraded vigilance and reaction time during simulated multi-day fire suppression under restricted sleep [5,6].

The early feasibility of implementing nationwide diagnostic procedures is a central strength of this cohort. The successful cascade from screening to treatment initiation of OSA demonstrates clinical engagement and operational scalability, which are elements often missing in occupational sleep research. Future analyses will evaluate treatment adherence, occupational outcomes, and cardiometabolic trajectories.

Limitations

This report presents preliminary baseline data from an ongoing prospective cohort and is therefore descriptive in nature, without inferential or multivariable analyses. As such, no associations or causal relationships can be established. Participation was voluntary and based on national dissemination, which may introduce selection bias, as individuals experiencing sleep-related symptoms may have been more likely to enroll. The total number of firefighters exposed to the invitation could not be precisely quantified, limiting the estimation of response rates. Several variables, including operational sleep metrics and comorbidities, were self-reported and may be subject to recall bias or misclassification. Although diagnostic testing followed structured criteria, detailed device-level reproducibility may vary across clinical settings. Finally, baseline findings do not permit evaluation of long-term clinical outcomes, treatment adherence, or occupational impact, which will be addressed in future longitudinal follow-up analyses.

Future research directions

Planned analyses include longitudinal assessment of PAP adherence, evaluation of occupational outcomes, and follow-up of cardiovascular and metabolic markers. Further investigations will examine operational sleep modeling and comparative benchmarking with international firefighter cohorts.

## Conclusions

Preliminary baseline data from the Associação Chama Saúde initiative demonstrate a high burden of sleep-related symptoms, increased screening-identified risk of OSA, and marked operational sleep restriction among Portuguese firefighters. The structured pathway from screening to diagnosis was feasible at a national level, supporting the broader integration of sleep health strategies within fire services. Longitudinal follow-up will be required to evaluate clinical impact and adherence to treatment.
